# Health advocacy and myopia prevention: a case description of what has been done and could be done to contain the myopia epidemic in children

**DOI:** 10.3389/fpubh.2026.1955463

**Published:** 2026-10-09

**Authors:** Vasanthi Iyer, Caroline C. W. Klaver, Sijmen A. Reijneveld

**Affiliations:** 1 Department of Health Sciences, University Medical Center Groningen, University of Groningen, Groningen, Netherlands; 2 Department of Child Health, TNO, Leiden, Netherlands; 3 Department of Ophthalmology, Erasmus Medical Center, Rotterdam, Netherlands; 4 Department of Epidemiology, Erasmus Medical Center, Rotterdam, Netherlands; 5 Department of Ophthalmology, Radboud University Medical Center, Nijmegen, Netherlands; 6 Institute of Molecular and Clinical Ophthalmology Basel, Basel, Switzerland

**Keywords:** eye health, health advocacy, myopia, public health, youth, activities

## Abstract

**Introduction:**

Myopia is becoming an epidemic among children globally, due to increased near screen exposure and reduced outdoor activity. Health advocacy—actions designed to gain political commitment, policy support, and social acceptance for health goals—can help to address this public health challenge. This paper reflects on health advocacy activities undertaken to contain the myopia epidemic and on potential future directions.

**Methods:**

We mapped activities using the American Heart Association (AHA) advocacy framework undertaken in the Netherlands from 2018 to 2026 via the innovative multidisciplinary network ‘View Outdoors’ (2018), targeting professionals, policymakers, and the public. We used the University of British Columbia (UBC) health advocacy framework perspective to assess the approaches, gaps, and opportunities for the next phase of the movement.

**Results:**

Using the AHA framework, the activities of the network target policy, systems and environmental change through factsheets, national guidelines, and multisector partnerships. The UBC-framework shows that the activities comprise both agency (working within existing structures) and activism (seeking to change structures), supporting the notion that sustainable public health change requires action across multiple sectors rather than isolated initiatives. Gaps include little structured involvement of youth and parents, and dependance on a small group of champions, creating vulnerability and a risk of bias. Attention to determinants such as social inequalities and financial barriers to spectacles create opportunities for strengthening the movement in the next phase.

**Conclusion:**

Health advocacy, through multisectoral engagement, can help to contain the myopia epidemic. Taken together, application of the AHA and UBC frameworks show the strengths of the Dutch approach to regard the combining of a broad advocacy portfolio with collaborative activities that link evidence, professional practice, public messaging, and policy influence. Future advocacy actions should focus on participation of children and parents that are directly affected, so their experience and preferences directly shape the agenda and priorities of advocacy efforts. This future approach should pair its strong agency and activism with systematic evaluation of behavioral change and deeper equity-focused partnership, and should address upstream determinants (structural and social conditions) to realize its public health potential.

## Introduction

Myopia, or near-sightedness, is rapidly emerging as a significant public health challenge, with prevalence rates rising sharply among children and young adults (1). Myopia is a refractive error of the eye, just like hyperopia and astigmatism are, but rates of this eye anomaly are increasing rapidly among the younger generations in most middle- and high-income countries (1, 2). The global prevalence of myopia is anticipated to exceed 740 million children and adolescents by 2050 (3). The determinants of myopia are multifaceted, involving both genetic, environmental and lifestyle factors, with determinants playing a role in childhood in particular, the period in which the eye develops and grows (4). Environmental factors are core contributors to the occurrence of myopia, in particular near work activities (screen-use, such as smartphones and computers) including reading books, and lack of being outdoors (5, 6). Being outdoors is a critical protective factor against myopia (6). High light intensity outdoors, with exposures up to over 100,000 lux on sunny days compared to indoor levels of 500 lux or less, is believed to stimulate dopamine release in the retina, which inhibits eye growth (5, 7). Encouraging children to spend at least 2 hours outdoors daily can significantly reduce the risk of developing myopia. Most environmental issues related to screen use and reduced outdoor play are fundamentally behavioral, shaped by the actions and attitudes of both parents and children (8). Interventions are needed to foster healthier routines and developmental outcomes for children. In the Netherlands, myopia has however not yet been identified as a public health problem. Advocacy to minimize the risk factors for myopia onset is considered the key by worldwide vision institutes (6, 9).

Health advocacy is defined by the World Health Organization as “a combination of individual and social actions designed to gain political commitment, policy support, social acceptance and systems support for a particular health goal or programme” (10). Health advocacy is also a core competence according to the CanMEDS physician framework, describing their abilities to improving the health and well-being of communities and the population as a whole (11). While myopia is relevant to all physicians, it is of particular significance for those active in public health. The Dutch Preventive child and youth healthcare (PCYH) is a unique, publicly funded system that provides well-childcare from birth to 18 years, thereby promoting the health and development of children. It stands as a cornerstone of public health and is shaped by a long tradition of universal access, early interventions, and multidisciplinary collaboration (12, 13). Vision screening is one of the core tasks of Dutch PCYH, currently entailing early detection of amblyopia, as determinant of visual impairment at a later age. Screening for myopia, as a refractive error of the eye, has not been defined as core task of the PCYH currently (14).

In the Netherlands, organized attention for myopia as emerging major public health challenge only started in 2018, when the multidisciplinary professional network ‘View Outdoors/ Focus on Outdoors’ (‘Zicht op Buiten’ in Dutch) had its first meeting. This was the start of a health advocacy movement focusing on myopia. The primary focus of the movement was to increase myopia awareness among professionals and policy makers, an important step in bringing about sustainable change in behavior (10). Behavioral change as such is a long and dynamic process (15, 16). The aim of this paper was to describe the health advocacy activities undertaken by the View Outdoors network to contain the myopia epidemic in the Netherlands and to identify opportunities for further myopia-prevention advocacy.

## Methods

### Setting/sample

We conducted a retrospective, descriptive, single-case study of health-advocacy activities undertaken by the View Outdoors network between 8th of June 2018 and 22^nd^ of April 2026. The case comprised the network and advocacy actions undertaken collectively by the network members or by individual members or small groups based on the network’s mission. The network is described as a case description in the Box 1.

The unit of analysis was an advocacy activity, defined as a time-bound action, product or event intended to influence professionals, policymakers, and/or the public in relation to myopia prevention. Activities included policy documents, guideline developments, scientific publications, educational materials, training events, media appearances, parliamentary engagement, lobbying, and coalition activities. Routine clinical work, informal discussions without a documented advocacy purpose and activities without sufficient description were excluded.

Data sources regarded the archive of the network, publicly available web materials, and documentary evidence supplied by core network members to compile a descriptive activity inventory.

### Procedure/measures

The first author searched all data sources to identify activities. Next, all identified activities were coded using two frameworks. First, we used the American Heart Association (AHA) advocacy framework to map the activities undertaken by the whole multidisciplinary network and/or by its individual/small group members. Second, we coded them using the University of British Columbia (UBC) health advocacy framework perspective to assess options for extending activities by comparing the types of actually undertaken activities with those that could have been undertaken (17). We describe the two frameworks hereafter.

### American Heart Association (AHA)-framework

The AHA advocacy approach links evidence and policy research to policy development, advocacy campaigns, media and coalition activities, lobbying, legislation, policy implementation, and outcome evaluation (18, 19). The framework has been designed to assess how advocacy efforts influence the social, political, and economic environments that affect health outcomes. To categorize activities, we used policy development, research findings, advocacy campaigns, and lobbying. To this we added two domains: communication and training, and social and other media to capture prominent activities in the case materials. We did not include legislation/regulation finalized because no relevant legally binding Dutch legislation has been finalized. Moreover, we excluded the analysis domains because the study did not assess policy implementation or outcomes. The resulting six categories were therefore an author-adapted, AHA-informed descriptive framework rather than a complete application of the original AHA framework. We distinguished two levels of provision of activities: (1) member-led activities, undertaken by individual members or small groups demonstrably connected to the View Outdoors mission or network; (2) collaborative activities, co-developed or co-delivered with one or more external organizations.

### University of British Columbia (UBC)- health advocacy framework

The UBC-framework maps activities along two intersecting axes: directed–shared and agency–activism, yielding four quadrants: directed–agency, directed–activism, shared–agency, and shared–activism (17), see Table 1. Directed agency regards actions in which health professionals primarily identify health needs and help patients, parents or communities access information or support within existing systems. Directed activism regards actions in which health professionals primarily identify health needs and priorities for change and advocate for sustained changes in practices, policies, or legislation. Shared agency regards activities in which patients, parents or communities identify or co-identify needs, and health professionals work with them to access or disseminate information, or support within existing systems. Shared activism involves activities in which patients, parents or communities identify or co-identify priorities for change and work with health professionals to reshape practices, policies, or legislation.

**Table 1:** Quadrants of the UBC-framework approach.

Advocacy approach: who identifies health needs and priorities for change?	Agency (working within existing systems)	Activism (changing practices, policies, or systems)
Directed (health professionals identify needs and advocate for patients, parents, communities)	Directed agency	Directed activism
Shared (patients, parents, communities identify or co-identify needs and priorities; health professionals advocate with them)	Shared agency	Shared activism

Based on the UBC-framework, the activities were divided into four categories: (1) Directed agency (2) Directed activism (3) Shared agency, and (4) Shared activism.

### Positionality statement

Two authors participated in the View Outdoors network and/or contributed to the advocacy activities described. This insider knowledge supported contextual understanding and identification of relevant materials but may also have influenced activity selection and interpretation. To address this potential intellectual and insider bias, we based the case description on available documentary sources, recorded sources in an activity inventory, distinguished documented activities from informal recollections, and used the AHA and UBC frameworks as explicit interpretive lenses.

### Analysis/reporting

We used a descriptive framework-based case analysis without formal qualitative coding. Activities were organized in relation to the advocacy domains derived from the AHA-framework and the way of participation of network partners, i.e., either as individual members or small groups, or as the whole network. Second, we categorized the activities according to the different approaches based on the UBC-perspective. Finally, we discussed to which degree further activities could be taken in specific categories of either of these two frameworks.

## Results

The network is described as a case description, see the Box 1.

BOX 1 **Case description: view outdoors (in Dutch, ‘Zicht op Buiten’)**

*Context*

In 2018, the professional network ‘Zicht op Buiten/View Outdoors’ was launched by a public child and youth health physician (PCYH) who was AJN (Dutch Association of PCYH physicians) ambassador Vision Insight (in short: ambassador). At its start, the network consisted of an ophthalmologist-epidemiologist, optometrists, orthoptists, PCYH professionals, pediatricians, a youth nurse, an orthopedic surgeon, a movement scientist-epidemiologist, health scientists, and psychologists. The network has been registered as a non-profit foundation in 2025.

*Mission*

The mission of the network is to promote outdoor physical activity and reduce sedentary behavior among children, thereby preventing myopia and associated health problems. The activities of the network focus on three target groups: (health) professionals, policy makers and public (parents of children). In the context of myopia, advocacy aims to:

- Influence policy development founded on research findings.

- Make strategic use of communication training and social media.

- Encourage collaboration for advocacy campaigns and lobbying.

*Advocacy activities*

Since 2018, this network has been promoting lifestyle changes among youth, specifically addressing the increase in screen use. It has grown into a movement involving more than 100 different professionals and organizations, including parent/patient/community organizations. To address the consequences of insufficient physical activity and time spent outdoors on youth health, the network has been focusing on areas such as vision, motor skills, posture, weight, sleep, and psychosocial health. The network has been undertaking various health advocacy activities to minimize the risk factors for excessive screen use and engaging in alternative solutions like more outdoor play and incorporate measures following the 20-20-2 rule (after 20 min of screen use, 20-s breaks, and 2 h outdoors daily) (5).

*Support and awards*

The network meets twice a year. It is financially supported by network partners such as the Oogfonds (a Dutch community trust dedicated to preventing and treating eye diseases, as well as supporting research and raising awareness about eye health) and Jantje Beton (a charitable trust that aims to create and encourage more play opportunities and outdoor spaces for children). In 2023, the network was awarded the Dutch PCYH-prize “Flora van Laar”, because of its activities.

*Description of activities per domain and ways of participation* (Table 2).

**Table 2:** Description of health advocacy activities per domain and ways of participation.

Health advocacy activities	Participation in the activity (X)		Targeted groups (X)			UBC-quadrant
	Individual members or small groups of network partners	Multi-partner network	Professionals	Policymakers	Public	
Policy development						
Factsheet excessive digital ‘near work’ usage, updates (2018, 2019, 2024)		X	X	X		Directed agency
Viewpoint on excessive digital ‘near work’ usage, updates (2018, 2025)	X		X	X		Directed agency
White Paper (2020)		X	X	X		Shared activism
Consensus guideline healthy screen use (2025)	X		X			Shared activism
Guidelines on myopia in childhood (2025)	X		X			Shared activism
Research findings						
Responsible use of screens: recommendations to limit myopia in children and young people. *Nederlands Tijdschrift voor Geneeskunde 2020; 164: D4106. [in Dutch]*	X		X			Directed agency
Outdoors of course! *TSG Tijdschrift voor Gezondheidswetenschappen 2021; 99: 125–127. [in Dutch]*	X		X	X		Shared agency
Parental behavior and near screen use in childhood: a route to reduce screen induced myopia. *Frontiers in Public Health 2025; 13: 1621687.*	X		X			Directed activism
Myopia and screen time in children: epidemic proportions. *European Journal of Public Health 2025; 35: 809–810*	X		X			Directed activism
Myopia and screen time: The Dutch method in addressing the epidemic with effective strategies. *European Journal of Public Health 2025; 35: 812–813.*	X		X			Directed activism
Communication and training						
Health organizations	X		X			Shared agency
- Lectures for general practitioners, pediatricians (national and international), insurance doctors, medical students, child mayors etc.	X		X		X	Directed agency/Shared agency
Educational discussions/campaigns						
- Webinars		X	X		X	Shared agency
- Oogfonds campaigns (2021-2022-2023-2024)	X		X		X	Shared agency/Shared activism
- Comprehension of the 20-20-2 rule for schools, a reading method (2024)		X	X		X	Shared agency
- Meetings with parents (2023)	X				X	Directed agency/ Shared agency
Multidisciplinary meetings						
- Network meetings twice a year		X	X			Shared agency
- Conferences (2023, 2025)		X	X		X	Shared agency
Social and other media						
Social/digital media						
- Podcast on myopia (2023)	X		X		X	Directed activism
- Newspaper articles, newsletters, websites, television programs (2024–2025), interviews for different magazines	X				X	Directed activism/Shared activism
Advocacy campaigns/Lobbying						
Campaign ‘eradicate myopia in the world’, and meeting with the Queen of the Netherlands	X		X		X	Directed activism/Shared activism
Political influence						
- Parliament member(s) involvement (2024, 2025) regarding the 20-20-2 rule/digital safety	X		X	X		Shared activism
- Multisectoral collaboration		X		X	X	Shared activism

See also the Supplementary table for a descriptive inventory of advocacy activities identified in the View Outdoors case materials, 2018–2026.

### Policy development

Activities addressing policy development started with a viewpoint on digital ‘near work’ usage (based on a factsheet launched by the network), made by a small committee involving a pediatrician, PCYH nurse and the ambassador, after which other activities addressing this soon followed (20). These are examples of directed agency, explaining the state-of-the-art situation regarding lifestyle. In November 2020, a White Paper was presented to the Secretary-General of the Ministry of Health, Welfare and Sport (VWS) to draw attention to the health consequences of insufficient outdoor activity and exercise among children and adolescents, with emphasis on vision, motor skills, posture, overweight, sleep, and psychosocial health. The paper included robust lifestyle recommendations, such as the 20-20-2 rule (after 20 min of screen use, a 20-s break, and 2 h outdoors daily) and the importance of at least 2 hours outdoors daily (5). The document was a call to action for policymakers, parents, schools, and health professionals to collaborate aiming for a healthier lifestyle for children. Contribution to the development of national guidelines was and is a process requiring collaboration with professional bodies and stakeholders. Two key guidelines, one concerning healthy screen use (completed, based on multi-stakeholder consensus including parents, health professionals, and political representatives) and the other targeting prevention of progressive myopia in children (nearing completion), reflect multidisciplinary and evidence-driven processes (21). The White Paper and guidelines are examples of shared activism, aimed at reshaping policies and legislation.

### Research findings

Various research findings were published for a large audience including researchers and other professionals. This resulted in a thesis involving both ophthalmological and public health perspectives and combining the views of policy, practice, and research (22). Two papers are examples of directed and/or shared agency, three others including a European viewpoint regarding myopia directed activism.

### Communication and training

Different organizations (health—educational—welfare) were influenced by creating awareness among professionals of diverse backgrounds. Discussions with these partners resulted in increased awareness among the public involved and participation in lifestyle campaigns promoting eye health resulted in increased awareness among parent organizations and participation in the network meetings, both of which also may lead to wider impact. A news comprehension program on the 20-20-2 rule was introduced in the reading methods for school children, the process resulting in more knowledge among the education professionals involved (23). Regular network meetings with invited lecturers added to the knowledge of the network partners, creating new opportunities for collaboration. These are all examples of shared agency.

### Social and other media

Varying between podcasts to news items, several television programs involving the ambassador and other partners focused on the importance of lifestyle on youth eye health, as directed and or shared activism examples.

### Advocacy campaigns and lobby

Influence through politics marked the years 2024 and 2025, when parliamentary debates took place in the Dutch House of Representatives, advocating the 20-20-2 rule. Digital safety and rules regarding age limits for digital use were yet again other themes discussed with another parliamentary representative. Both activities were initiated by two small committees of network members, the first involving eye health professionals and the second including professionals involved with digital literacy (24). Illustrative multisectoral initiatives include working along with Vision 2020 Netherlands, advocating for spectacles to be recognized as an essential medical tool and a basic right for children (25). Another is the Rotary Healthy Kids Ooststellingwerf program, which encourages outdoor physical activity among children, including those with visual impairment; the Eye Fund 20-20-2 toolkit and educational material for primary schools; the Lions Outdoor Sports program for grade 7 pupils in Oudekerk aan de Amstel; and instructional sessions on children’s rights and eye health delivered to child mayors (26). All these activities fit into directed and/or shared activism.

## Discussion

The aim of this paper was to assess the health advocacy activities to contain the myopia epidemic which were undertaken by the network and assess opportunities for future directions. Applying the AHA-framework showed that the network activities were diverse, with the strongest emphasis on communication and training, policy development, and dissemination of research findings. In contrast, activities in the category ‘advocacy campaigns and lobby’ were applied the least. Direct influence on policymakers was thus used more selectively than public awareness and professional education. These findings suggest that successful public health advocacy does not rely on a single intervention, but on a combination of coordinated activities operating simultaneously at professional, policy, and public levels. This supports the notion that sustainable public health change requires action across multiple sectors rather than isolated initiatives (10, 15). Furthermore, this case description illustrates how a small multidisciplinary network may contribute to agenda setting for an emerging public health challenge. By combining scientific evidence with strategic advocacy, the network contributed to increasing professional and policy attention for myopia prevention. This illustrates an important principle of health advocacy: evidence alone rarely changes practice or policy. Translation of evidence into accessible messages, strategic partnerships and repeated communication are equally important for achieving societal impact.

Applying the UBC health advocacy framework demonstrated that advocacy goes beyond delivering health care alone, with individual/small group of professionals also engaging in policy networks and coalition building. The UBC-framework distinguishes between actions that work within existing systems (“agency”) and those that try to change systems (“activism”), at both individual and collective levels, showing that advocacy can range from direct help to structural transformation (17). Interestingly, few activities were confined to directed agency or directed activism alone. Instead, most evolved towards shared agency or shared activism, suggesting that broader partnerships became increasingly important as the movement matured. Gaps include little structured involvement of youth and parents and dependance on a small group of champions, creating vulnerability and a risk of bias.

### Implications: future directions

Building on these findings, future advocacy should focus on expanding participation beyond the current professional network. At present, a ban on smartphones at schools has been mandated, without legal or financial penalties, and there is a strong lobby for the introduction of social media age limits. A grass roots movement of parents to break peer pressure by coordinating collective action among parents involving schools is active and has joined the network (27). More active involvement of youth, parents, and schools could strengthen both the legitimacy and the reach of future advocacy activities. These broader partnerships may also strengthen the conditions for future policy development regarding healthy screen use and time spent outdoors. This would involve more time spent outdoors during school hours, an aspect which involves support from the Education department of the Dutch Government, emphasizing policy makers as target group. A growing focus on health equity, including discussions on equal opportunities for all children and potential reimbursement of spectacles can help to reduce socioeconomic and access-related disparities in visual health. Equal opportunities for all children and national health insurance compensation for spectacles involves again policy makers as target group (24).

Our analyses also show several strengths of the current HA approach that we can employ further, and several limitations that could be improved. A major strength of the current advocacy portfolio lies in its breadth and coherence, operating at various levels, spanning both agency and activism at all levels. That should be the basis to build upon in any further approach.

The analyses also show several limitations of the current approach. An important limitation is that the progress of societal changes was not monitored, neither was the impact of the activities on the public including behavioral change evaluated. Until now resources lacked to do so, with limited means invested in collaborative lobby efforts which yielded robust products like guidelines and publications. The AHA- and UBC-framework analyses showed a consistent pattern. Most activities focused on building awareness, strengthening professional collaboration and translating evidence into practice and policy, whereas relatively few activities involved direct political advocacy or directed activism. However, system level changes are needed to address health inequalities and health advocacy should become a more explicit role for all public health professionals (10, 28). Adaption in strategies may be needed as evidence increases; this requires continuous feedback loops and data-driven advocacy, with proper assessment of the behavioral impact.

A methodological limitation of this single case description is that although it provides valuable insights into advocacy processes, their generalizability to other countries and healthcare systems needs to be confirmed. Moreover, activities were identified and categorized retrospectively, which may have introduced bias despite the use of established theoretical frameworks.

## Conclusion

In conclusion, the practice of health advocacy activities as described regarded teamwork and personal activities, which aligns with theoretical considerations. Our analyses show that the network evolved from individual/small group advocacy initiatives towards multidisciplinary collaboration, using research to strengthen professional practice, build consensus and support policy development.

Although the behavioral impact of these activities was not evaluated, the breadth and reach of the activities suggest that they may have contributed to increased awareness of myopia prevention and strengthened efforts to promote protective lifestyle behavior. In general, methodologically the application of the AHA- and UBC-frameworks can help to elaborate the opportunities for strengthening of current practices. Specifically, regarding the myopia epidemic, further health advocacy could reduce the occurrence of myopia and improve lifestyle in general among children, i.e., create conditions for political commitment, policy support, social acceptance, and systems change. Future advocacy could build on the foundation laid by strengthening direct engagement with policymakers, broadening participation of children, parents, and schools, and systematically evaluating advocacy outcomes. Such an approach may further increase the networks’ contribution to reducing myopia and promoting healthy lifestyles among children.

## Data Availability

The original contributions presented in the study are included in the article/Supplementary material, further inquiries can be directed to the corresponding author.
