# Supplementary material for: Health advocacy and myopia prevention: a case description of what has been done and could be done to contain the myopia epidemic in children
Source: Front Public Health. 2026 Oct 9;14:1955463. doi: 10.3389/fpubh.2026.1955463 (PMC13735796; doi:10.3389/fpubh.2026.1955463)
Supplement: Supplementary file 1 [file Data_Sheet_1.pdf]

Supplementary Table 1: Descriptive inventory of advocacy activities identified in the View Outdoors case materials, 2018 - 2026

ID	Activity	Period	Source(s)	AHA-category/ categories	UBC-category	Rationale
1	Launch of the View Outdoors professional network	2018	Case description; network archive	Advocacy campaigns; communication and training	Directed agency	A multidisciplinary network was established to share knowledge, connect professionals and promote lifestyle-oriented myopia prevention through existing professional structures.
2	Development and publication of a factsheet on digital near work and lifestyle	2018 with updates in 2019 and 2024	Network archive; webpages of network partners	Research findings; communication and training	Directed agency	A network-informed evidence resource communicated evidence-based lifestyle and screen-use information to relevant audiences without directly seeking to change policy.
3	Viewpoint on digital near work use	2018 with update in 2025	Network records webpages of network partners;	Policy development ; research findings	Directed agency	A small committee of a paediatrician, p nurse and the ambassador used evidence to make a position statement on digital near work and child health.
4	Awareness-raising discussions with health, educational and welfare organizations	2018 onwards	Meeting records; partner correspondence; campaign records; network archive	Communication and training; advocacy campaigns	Shared agency	Network partners including Eye Funds, Jantje Beton and patient organizations engaged in awareness among other organizations and the public through discussion and collaboration to increase knowledge and participation within existing sectors and structures.
5	Regular multidisciplinary View Outdoors network meetings with invited lecturers	2018 onwards; twice yearly	Meeting agendas; programmes; attendance lists; presentations; network archive	Communication and training	Shared agency	Recurring meetings to build capacity, shared evidence and facilitated collaboration among network partners within professional structures. Eye Funds, Jantje Beton and patient organizations participated from late 2018 onwards.
6	News items on lifestyle and youth eye health	2018 onwards	News articles; media archive; network records	Social and other media;	Directed and/or	Media coverage amplified prevention messages and contributed to public and policy-oriented agenda setting.

ID	Activity	Period	Source(s)	AHA-category/ categories	UBC-category	Rationale
				advocacy campaigns	shared activism	Patient organizations and communities collaborated and contributed.
7	Participation in lifestyle campaigns promoting eye health	2018 onwards;	Campaign materials; partner websites; network archive; media records	Advocacy campaigns; communication and training; social and other media	Shared agency	As a multisectoral initiative members collaborated in public-facing campaigns to disseminate eye-health and lifestyle messages, mainly by mobilizing existing organizational and communication channels.
8	Network expansion and engagement of more than 100 professionals and organizations	2018 onwards	Membership/participation records; meeting lists; network archive	Advocacy campaigns; communication and training	Shared agency	Expansion of the network increased collaboration and capacity for disseminating prevention messages, although the membership growth itself should be distinguished from discrete advocacy activities.
9	White Paper on insufficient outdoor activity, exercise and child health presented to the Ministry of Health, Welfare and Sport	2020	White Paper; webpage of network; correspondence record; network archive	Policy development; advocacy campaigns; lobby	Shared activism	A policy-oriented, multisectoral document, initiated by the Eye Funds as a community trust that calls on policymakers, parents, schools and health professionals to act on outdoor activity, screen use and child health.
10	Promotion of the 20-20-2 rule through the White Paper and associated advocacy	2020 onwards	White Paper; factsheets; campaign materials; network archive	Communication and training; advocacy campaigns; policy development	Shared activism	The network promoted a specific preventive recommendation while seeking broad institutional and policy support for healthier screen-use and outdoor-time practices. Partners were communities like the Eye Funds and Jantje Beton (*The Dutch National Children's Play Trust)*

ID	Activity	Period	Source(s)	AHA-category/ categories	UBC-category	Rationale
11	Publication of two research papers characterized as agency-oriented	2020<br>2021	Peer-reviewed publications; author records; bibliographic databases	Research findings	Directed and/or shared agency	These publications disseminated scientific knowledge for professional and research audiences; the second paper was in collaboration with the Eye Funds.
12	Television/radio appearances by the ambassador and other network partners on lifestyle and youth eye health	2021, 2024, 2025	Television/radio programme records; broadcast clips; media archive; network records	Social and other media; advocacy campaigns	Directed and/or shared activism	Network representatives used mass media to reach the public and promote lifestyle and prevention messages that may challenge prevailing screen-use norms.
13	Podcasts addressing lifestyle and youth eye health	2022	Podcast episodes; hosting-platform records; network or participant records	Social and other media; communicati on and training	Directed and/or shared activism	Public-facing audio communication with contributions from different network partners including patients was used to advocate for lifestyle change and raise awareness of the relationship between outdoor activity, screen use and eye health.
14	Educational sessions for child mayors on children’s rights and eye health	2023-2026	Session materials; child-mayor programme records; network documentation	Communicati on and training; advocacy campaigns	Directed activism	The sessions informed young civic representatives about rights and eye health, with the potential to foster advocacy and norm change through youth participation.
15	Receipt of the Flora van Laar Dutch PCYH prize	2023	Award announcement; AJN/PCYH award record; network archive	Not coded as an advocacy activity; contextual recognition	Not applicabl e	The award documents external recognition of the network’s work but is an outcome or contextual marker, not an advocacy activity in itself.
16	Introduction of a news-comprehension programme on the 20-	2024	Educational programme material; published on school sites;	Communicati on and training	Shared agency	The intervention in collaboration with the Eye Funds and other partners disseminated preventive knowledge to children and education professionals through the existing school curriculum.

ID	Activity	Period	Source(s)	AHA-category/ categories	UBC-category	Rationale
	20-2 rule in primary-school reading methods		network/partner documentation			
17	Parliamentary advocacy for the 20-20-2 rule	2024–2025	Parliamentary debate records; parliamentary questions or motions; committee correspondence; network archive	Lobby; policy development ; advocacy campaigns	Directed activism	A small committee of eye-health professionals engaged political processes to advocate for a preventive lifestyle recommendation and potential structural change.
18	Political engagement on digital safety and age limits for digital use	2024–2025	Parliamentary representative correspondence; committee records; webpage archives; network archive	Lobby; policy development	Shared activism	Professional and parent organizations involved in digital literacy engaged with parliamentary representatives on regulatory and policy issues intended to influence children’s digital environment.
19	20-20-2 educational material for primary schools	2024 onwards	Eye Fund toolkit; school/partner record; network documentation	Communication and training	Shared agency	A multisectoral collaborative activity disseminating knowledge through existing educational structures without directly seeking policy change.
20	Rotary Healthy Kids Ooststellingwerf programme promoting outdoor physical activity	2024	Rotary programme record; local project materials; partner documentation; network records	Advocacy campaigns; communication and training	Shared activism	A local multisectoral initiative sought to shift community practice and opportunities for outdoor activity, including activities for children with visual impairment.
21	Lions Outdoor Sports programme for grade 7 pupils in Ouderkerk aan de Amstel	2024	Lions programme materials; school or municipal records; network/partner documentation	Advocacy campaigns; communication and training	Shared agency	The programme promoted outdoor physical activity among pupils through a community-school initiative, primarily operating within existing local structures.

ID	Activity	Period	Source(s)	AHA-category/ categories	UBC-category	Rationale
22	Development of the national guideline on healthy screen use	2025	Guideline-development documentation; participating stakeholder records	Policy development; research findings	Shared activism	A multistakeholder, consensus-based guideline involved parents, health professionals and political representatives and sought to influence practice at a system level.
23	Publication of three research papers, including a European viewpoint on myopia	2025	Peer-reviewed publications; European viewpoint; author records; bibliographic databases	Research findings; advocacy campaigns	Directed activism	The papers, particularly the viewpoint, go beyond reporting evidence by advocating changes in professional norms, prevention priorities or policy approaches.
24	Collaboration with Vision 2020 Netherlands on recognition of spectacles as an essential medical tool and children's right	2025	Vision 2020 Netherlands materials; joint advocacy documents; network/partner records	Advocacy campaigns; lobby; policy development	Shared activism	A multisectoral collaboration sought recognition of spectacles as an essential medical tool and a basic right for children, implying rights-based systems and policy change.
25	Registration of View Outdoors as a non-profit foundation	2025	Foundation registration; statutes; network archive	Not coded as a primary advocacy activity; organizational development	Not applicable	Legal registration strengthened the organizational infrastructure and may support future advocacy, but it is not in itself an activity directed at professionals, policymakers or the public.
26	Contribution to the guideline on prevention of progressive myopia in children	2026 Ongoing, close to completion	Guideline working-group records; network documentation	Policy development; research findings	Shared activism	Development of a professional guideline jointly with patient organizations y intending to influence prevention and clinical practice beyond individual encounters.
27	Thesis integrating ophthalmological and	2026	Institutional repository; related publications	Research findings	Directed agency	A research output disseminating evidence relevant to policy, practice and research; its primary route was knowledge generation and communication.

ID	Activity	Period	Source(s)	AHA-category/ categories	UBC-category	Rationale
	public-health perspectives on myopia					
